# 300-GHz Photonics-Aided Wireless 2 × 2 MIMO Transmission over 200 m Using GMM-Enhanced Duobinary Unsupervised Adaptive CNN

**DOI:** 10.3390/s26030842

**Published:** 2026-01-27

**Authors:** Luhan Jiang, Jianjun Yu, Qiutong Zhang, Wen Zhou, Min Zhu

**Affiliations:** 1 The State Key Laboratory of ASIC and System, Key Laboratory for Information Science of Electromagnetic Waves (MoE), School of Information Science and Technology, Fudan University, Shanghai 200433, China; 25113090098@m.fudan.edu.cn (L.J.); 22110720081@m.fudan.edu.cn (Q.Z.); zwen@fudan.edu.cn (W.Z.); 2 National Mobile Communications Research Laboratory, Southeast University, Nanjing 210096, China; minzhu@seu.edu.cn; 3 Purple Mountain Laboratories, Nanjing 211111, China

**Keywords:** photonic-aided terahertz transmission, multiple-input multiple-output, duobinary shaping, convolutional neural network, gaussian mixture model

## Abstract

**Highlights:**

**What are the main findings?**

- A GMM-enhanced duobinary unsupervised adaptive CNN (DB-UACNN) is proposed to enhance duobinary filtering without relying on training labels, enabling robust operation under unknown terahertz wireless channels.
- In a 300-GHz photonics-aided 2 × 2 MIMO wireless system over 200 m, the proposed scheme achieves up to 2.6 dB additional SNR gain over conventional duobinary filtering and maintains BER below the 7% HD-FEC threshold at 100 Gbit/s.

**What are the implications of the main findings?**

- The proposed label-free, GMM-guided learning framework removes the dependence on pilot symbols or ideal references, improving spectral efficiency and adaptability in practical THz wireless systems.
- Combining duobinary shaping, MLSD, and unsupervised neural enhancement offers a scalable DSP scheme for future high-capacity MIMO terahertz links, supporting beyond-100 Gbit/s transmission in bandwidth-limited and nonlinear environments.

**Abstract:**

Terahertz wireless communication offers ultra-high bandwidth, enabling an extremely high data rate for next-generation networks. However, it faces challenges including severe propagation loss and atmospheric absorption, which limits the transmission rate and transmission distance. To address the problem, polarization division multiplexing (PDM) and antenna diversity techniques are utilized in this work to increase system capacity without changing the bandwidth of transmitted signals. Meanwhile, duobinary shaping is used to solve the problem of bandwidth limitation of components in the system, and the final duobinary signals are recovered by maximum likelihood sequence detection (MLSD). A Gaussian mixture model (GMM)-enhanced duobinary unsupervised adaptive convolutional neural network (DB-UACNN) is proposed, to further deal with channel noise. Based on the technologies above, a 2 × 2 multiple-input multiple-output (MIMO) photonic-aided terahertz wireless transmission system at 300 GHz is demonstrated. Experimental results have proved that the signal-to-noise ratio (SNR) gain of duobinary shaping is up to 1.87 dB and 1.70 dB in X-polarization and Y-polarization. The proposed GMM-enhanced DB-UACNN also shows extra SNR gain of up to 2.59 dB and 2.63 dB in X-polarization and Y-polarization, compared to the conventional duobinary filter. The high transmission rate of 100 Gbit/s over the distance of 200 m is finally realized under a 7% hard-decision forward error correction (HD-FEC) threshold.

## 1. Introduction

With the gradual maturity of fifth-generation (5G) communication technologies, the sixth-generation (6G) mobile communication, which targets higher frequency bands and wider bandwidth, has been globally focused on as a research hotspot [1]. Among candidate technologies, terahertz (THz) communication is considered as a key technique for future ultra-high-speed and ultra-large-capacity wireless communication systems, for its extremely high carrier frequency range (0.3–10 THz) and ultra-broad available spectrum resources [2,3,4]. On this basis, photonic-assisted technology generates THz signals through the photonic process in THz wireless communication, and it has offered advantages such as wide modulation bandwidth, high frequency tunability, and huge system flexibility [5,6]. It has been verified that signals of various modulation formats have been successfully demonstrated in photonic-assisted wireless communication systems across multiple frequency bands [7,8,9,10,11].

However, in most photonic-aided wireless systems, the limited SNR at the receiver end results in limited performance [12,13,14]. To solve the problem, PDM and antenna diversity techniques can be employed to double the system capacity without changing the bandwidth of signals [15,16]. Moreover, the antenna diversity technique can also help improve SNR [17]. In 2021, Pham et al. demonstrated a 3 × 3 MIMO radio-over-fiber (RoF) system in the W-band. By integrating WDM and PDM technologies with orthogonally polarized MIMO antenna systems, the system capacity was enhanced to approximately 132 Gb/s, and the spectral efficiency was improved to 10.2 bit/s/Hz [18]. In 2024, Cai et al. replaced the traditional 2 × 2 MIMO structure with a dual-polarized SISO link, achieving a transmission rate of 504 Gb/s over a 20 km fiber and 1.3 m wireless link in the W-band [19]. In 2024, Zhang et al. implemented a photonic-assisted 2 × 2 dual-polarization W-band MIMO wireless system. By employing MRC and other advanced digital signal processing (DSP) techniques, the system achieved up to a 7.1-dB SNR gain over a 1 × 1 link, with an additional 2.9 dB gain from dual-polarization [20].

Furthermore, many studies have proved that advanced THz devices can greatly improve transmission performance [21]. In 2021, Moon et al. constructed a THz wireless link using a 17_dBm uni-traveling carrier photodiode (UTC-PD) at the transmitter and a Schottky barrier diode as detector at the receiver. The system successfully transmitted a 90-Gb/s PAM-8 signal over a wireless distance of 1.4 m with a bit error rate (BER) below the 20% soft-decision forward error correction (SD-FEC) threshold [22]. In 2025, Li et al. reported modified uni-traveling-carrier photodiodes achieving an ultra-wide bandwidth of 206 GHz together with a high external responsivity of 0.81 A/W, enhancing the performance of photonic-enabled sub-THz and THz transmitters [23].

Nevertheless, the performance of THz wireless communication systems can be severely degraded due to signal noise and the non-ideality of components [24,25]. Several studies concentrate on reducing the negative influences of underlying problems, including non-uniform spectral power distribution of laser sources, uneven frequency response of each amplifier, and limited system bandwidth [26,27,28]. These factors can lead to waveform distortion in time-domain and uneven frequency-domain response, with high-frequency signal attenuation being especially typical [29]. Previous studies commonly use two methods to address such issues. The first is pre-emphasis, which amplifies high-frequency components at the transmitter to compensate for channel losses [30]. However, in dynamic channels, this technique lacks adaptability and accuracy facing a nonlinear effect and time varying nature [31,32]. Alternatively, adaptive equalization algorithms are effective. Yet while compensating for high-frequency losses, equalizers also amplify high-frequency noise inevitably, thereby limiting the SNR performance [33]. Thus, adaptive dynamic wireless channel estimation and noise compression in received signals are promising ways to further improve the SNR.

In recent years, the integration of neural networks into DSP has demonstrated remarkable potential in addressing challenges in nonlinearity. Unlike approaches that rely heavily on prior analytical assumptions, neural networks offer strong data-driven adaptability and nonlinear modeling capabilities. This makes them particularly effective in scenarios involving channel distortion and symbol misalignment. In 2022, Bluemm et al. reported using duobinary-trained neural network equalization to achieve 200 Gb/s IM/DD PAM4 over 10 km [34]. In 2025, Wang et al. developed a MIMO neural network combined with maximum-likelihood phase recovery for transoceanic coherent optical transmission, focusing on phase noise mitigation in ultra-long-haul fiber links [35]. The existing works have already validated the feasibility of NN equalizers in DSP and the advantages of duobinary training target, but no prior work has used CNNs to enhance classical duobinary filters, while initializing network weights from the classical duobinary (DB) filter. Also, employing GMM outputs as pseudo-labels to eliminate the need for dedicated training data is rarely used in this area. A literature survey confirms this approach is novel and fills a unique gap in neural-equalizer design.

In this work, a 2 × 2 MIMO 300-GHz wireless THz transmission system based on PDM and antenna diversity multiplexing is constructed, as reported in [36,37,38]. To mitigate high-frequency attenuation, we integrate duobinary shaping [39]. Also, a GMM-enhanced duobinary unsupervised adaptive convolutional neural network (DB-UACNN) is proposed. Unlike conventional works that either rely purely on analytical receiver models or fully data-driven neural equalizers, this work aims to bridge the gap between model-based signal processing and unsupervised neural enhancement in bandwidth-limited wireless systems. As a result, a net data rate of 100 Gbit/s is achieved for QPSK signals over a 200 m wireless link with BER stays below 7% HD-FEC threshold. The proposed scheme is particularly suited for fixed wireless backhaul and short-range fronthaul scenarios at THz frequencies, where line-of-sight transmission and high data-rate requirements are dominant, enabling receiver-side enhancement without additional signaling overhead.

The remainder of this article is organized as follows. Section 2 introduces the principles of duobinary shaping, MLSD, and the optimized DB neural networks. Section 3 describes the 300-GHz 2 × 2 MIMO THz wireless communication system and the associated DSP architecture. Section 4 demonstrates the experimental performance of the proposed DSP algorithms. Section 5 gives the conclusion and prospect of the paper.

## 2. Principles of Receiver Digital Signal Processing

### 2.1. Duobinary Shaping

Duobinary shaping is introduced to compress high-frequency components [33]. Specifically, several reports have been made by using duobinary shaping as a post-filter of DSP at the receiver [33,40]. The impulse response of the DB filter is given as the following:

(1) h(n)=δ(n)+δ(n−1)

Assume that the QPSK signal is expressed as, and the derivation of the process above is as follows:

(2) y(n)=x(n)∗h(n)=x(n)+x(n−1)

**Figure 1:**
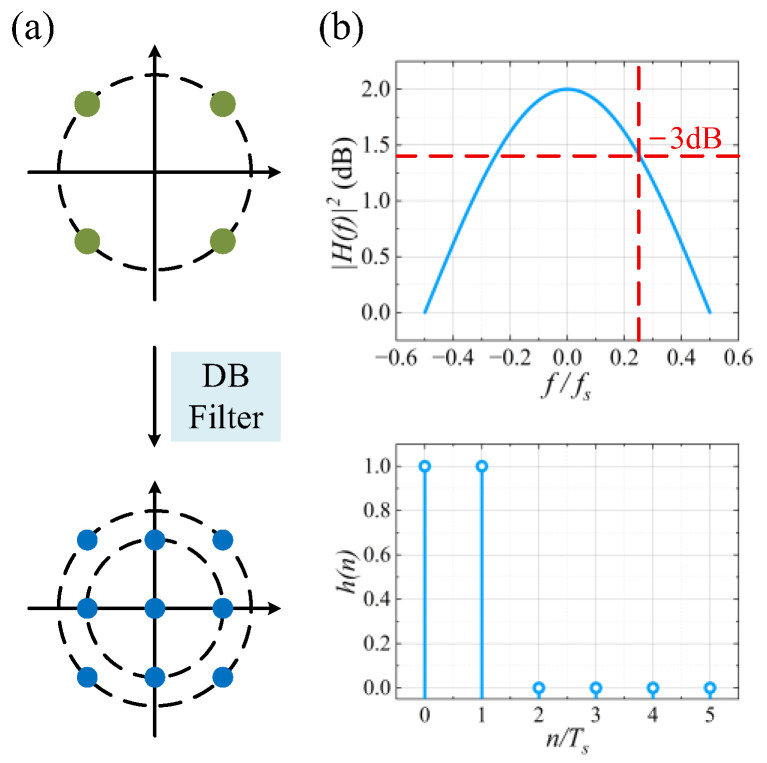
(**a**) Constellation diagrams before and after duobinary shaping, and (**b**) frequency response and impulse response of the low-pass filter.

From (2), the induced ISI creates a correlation between adjacent symbols and forms duobinary characteristics of a 3-level amplitude of 0, +1, −1. as shown in Figure 1a. The frequency response of the DB filter is expressed as:

(3) $$H(e^{j\omega })=1+e^{-j2\pi f}$$

In the frequency domain, it results in bandwidth compression. As shown in Figure 1b, the 3-dB bandwidth of the filter is half of $f_{s}$, which symbolize the signal baud rate. Suppose that the bandwidth-limited signal after linear equalizers is

(4) r(n)=s(n)+w(n)

s(n) and w(n) are the expected signal and noise. The power spectral densities are $S_{s}(f)$ and $S_{w}(f)$. To avoid gain scaling, the filter is normalized to unit energy:

(5) $$h_{\mathrm{DB}}[n]=\frac{1}{\sqrt{2}}[1,1]$$

The frequency response and squared magnitude response are

(6) $$\begin{array}{l} H_{\mathrm{DB}}(e^{j2\pi f})=\frac{1+e^{-j2\pi f}}{\sqrt{2}}=\sqrt{2}e^{-j\pi f}\cos(\pi f)\\ g(f)≜\;|H_{\mathrm{DB}}(e^{j2\pi f}){|}^{2}=2\cos^{2}(\pi f) \end{array}$$

where g(f) is a low-pass weighting function that attenuates the high-frequency band. Without DB post-filtering, the SNR at the equalizer output is

(7) $$\mathrm{SNR}=\frac{\int_{-f_{s}/2}^{f_{s}/2}S_{s}(f)df}{\int_{-f_{s}/2}^{f_{s}/2}S_{w}(f)df}.$$

With DB post-filtering, the signal and noise powers are both weighted by g(f):

(8) $${\mathrm{SNR}}_{\mathrm{DB}}=\frac{\int_{-f_{s}/2}^{f_{s}/2}g(f)S_{s}(f)df}{\int_{-f_{s}/2}^{f_{s}/2}g(f)S_{w}(f)df}.$$

Taking the ratios and comparing the SNRs of DB-filtered signals and original signals:

(9) $$\frac{{\mathrm{SNR}}_{\mathrm{DB}}}{\mathrm{SNR}}=\frac{\int_{-f_{s}/2}^{f_{s}/2}g(f)S_{s}(f)df}{\int_{-f_{s}/2}^{f_{s}/2}S_{s}(f)df}/\frac{\int_{-f_{s}/2}^{f_{s}/2}g(f)S_{w}(f)df}{\int_{-f_{s}/2}^{f_{s}/2}S_{w}(f)df}.$$

Define normalized spectral weights

(10) $$w_{s}(f)=\frac{S_{s}(f)}{\int_{-f_{s}/2}^{f_{s}/2}S_{s}(f^{\prime })df^{\prime }},\;\;w_{w}(f)=\frac{S_{w}(f)}{\int_{-f_{s}/2}^{f_{s}/2}S_{w}(f^{\prime })df^{\prime }}$$

Then the result becomes a ratio of expectations:

(11) $$\frac{{\mathrm{SNR}}_{\mathrm{DB}}}{\mathrm{SNR}}=\frac{E_{w_{s}}[g(f)]}{E_{w_{w}}[g(f)]},\;E_{w_{x}}≜\int g(f)w_{x}(f)df$$

Since g(f) is strictly decreasing with frequency, as the signal spectrum is more concentrated at low frequencies due to the bandwidth effect in high-frequency attenuation, while the noise spectrum after equalization is more concentrated at high frequencies (due to noise amplification at band edges), then

(12) $$E_{w_{s}}[g(f)]>E_{w_{w}}[g(f)]\;\;\Rightarrow \;\;{\mathrm{SNR}}_{\mathrm{DB}}>\mathrm{SNR}$$

Hence, duobinary shaping has been widely applied in Nyquist WDM and bandwidth limited systems, and also effective in high-frequency limitation [41,42].

### 2.2. Maximum-Likelihood Sequence Detection

In duobinary shaping, strong filtering of the signal can be regarded as an encoding operation. In this work, MLSD is applied as the decoder of duobinary-shaped QPSK signals [43]. By maximizing the posterior probability of the received sequence, MLSD achieves optimal estimation of the transmitted symbol sequence.

MLSD is based on Bayesian criteria [44]. Given that the transmitted and received signals are $s_{n}$ and $r_{n}$, respectively, the criterion can be equivalently expressed as minimizing the Euclidean distance between $r_{n}$ and channel-transmitted $s_{n}$:

(13) $$\begin{array}{ll} \overset{⌢}{s} & =\arg\max P(r\;|\;s)\\ & =\arg\sum_{n=1}^{N}|r_{n}-H_{channel}s_{n}{|}^{2} \end{array}$$

where $H_{channel}$ is the channel function. In order to decrease the computing complexity, MLSD is typically implemented using the Viterbi algorithm [45,46].

### 2.3. Optimized DB Neural Networks

Joindot et al. have proved that duobinary shaping combined with optimal receiver filtering (e.g., MLSD) achieves the best possible performance under Gaussian noise conditions [47]. Consider the received signal according to (4), after filtered with classical DB filter, duobinary signals go with:

(14) $$r_{DB}(n)=[x(n)+x(n-1)]+[w(n)+w(n-1)]$$

If $w(n)∼N(0,\sigma^{2})$, the output noise [w(n)+w(n−1)] remains Gaussian as the classical DB filter is a linear system. As a result, it will not introduce structural degradation in AWGN channels. However, considering the received signals under non-Gaussian white noise, like nonlinear noise as follows:

(15) $$w(n)=k_{1}x(n)+k_{2}x^{2}(n)+\eta (n)+O(x^{3}(n))$$

where η(n) is the additive noise. Higher-order terms are collectively denoted by the asymptotic notation $O(x^{3}(n))$. Since such terms typically contribute marginally in moderate signal power scenarios, they are omitted in subsequent modeling and analysis. Then (14) is rewritten as:

(16) $$r_{DB}(n)=(1+k_{1})[x(n)+x(n-1)]+k_{2}q(n)+\tilde{\eta }(n)$$

As $q(n)=x^{2}(n)+x^{2}(n-1)$, $\tilde{\eta }(n)=\eta (n)+\eta (n-1)$. Thus, classical DB filters produce biased and nonlinear outputs, which are correlated to parameters $k_{1}$ and $k_{2}$, respectively. The squared terms $x^{2}(n)$ introduce signal-dependent offsets into the detection process, resulting in degraded decision accuracy, thus decreasing the MLSD robustness.

**Figure 2:**
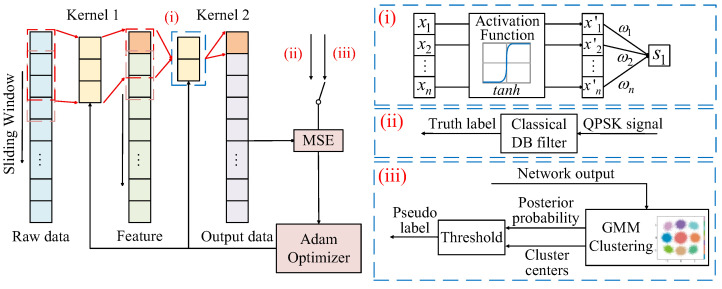
Principle of DB-CNN models in both supervised term and GMM-enhanced unsupervised term, where subfigure (**i**) symbolizes the nonlinear activation process, (**ii**) represents the ground-truth label of supervised DB-CNN, and (**iii**) means the soft-label in DB-UACNN.

As a result, conventional DB filtering becomes suboptimal under unrealistic propagation. We propose a neural network enhanced DB filters to break the limitation illustrated above. The proposed DB-based two-layer CNN models are demonstrated in Figure 2. The input signal is unfolded through a sliding window. The CNN adopts a two-layer structure. In the first layer, the kernel is initialized as the classical duobinary filter as $h_{Kernel1}=[1,1,0,\ldots ,0]$ to ensure a more stable training by physical prior information. As is shown in Figure 2i, a Tanh activation function introduces nonlinear response characteristics. The second layer is initialized as $h_{Kernel2}=[1,0,0,\ldots ,0]$ to help enhance the filtering response. This model-based initialization provides a meaningful physical prior and ensures that the network starts as conventional DB filtering. Such a prior is particularly important for unsupervised learning. Without DB initialization, the network output may fail to form the expected 9-point duobinary structure, and the GMM becomes unreliable due to unstable clustering, which may lead to inaccurate pseudo-labels and degraded con-vergence.

In Figure 2ii, the training procedure is based on supervised learning, where the principal objective is to minimize the mean squared error (MSE) loss between the filtered output and the target signal. The target signal is the expected duobinary signal, which is obtained by the expected QPSK signal filtered by the classical DB filter. The Adam optimizer is used to update the network parameters, facilitating joint modeling and compensation of both the signal structure and non-ideal channel effects.

However, the supervised DB-CNN relies heavily on ideal transmitted symbols as ground-truth during training. This reliance strongly restricts the net data rate and reduces its effective spectral efficiency, as it sacrifices part of the data as the training set. In scenarios such as unavailable expected symbols or symbol timing mismatch, the performance of supervised methods degrades significantly.

Hence, as demonstrated in Figure 2iii, the optimized GMM-enhanced DB-UACNN adopts an unsupervised learning strategy, which utilizes GMM cluster assignments to generate pseudo-labels. GMM approximates the target distribution as a mixture of multiple Gaussian components. The probability density function of GMM is defined as a weighted sum of multiple Gaussian distributions:

(17) $$p(x)=\sum_{i=1}^{K}\alpha_{i}\cdot N(x|\mu_{i},\Sigma_{i})$$

where K is the number of Gaussian components. $\alpha_{i}$ is the mixing coefficient of the $i^{th}$ cluster, which satisfies $\sum \alpha_{i}=1$. $\mu_{i}$ represents the mean vector and $\Sigma_{i}$ means the covariance matrix of the $i^{th}$ cluster. $N(x|\mu_{i},\Sigma_{i})$ represents the multivariate Gaussian distribution, formulated as:

(18) $$N(x|\mu_{i},\Sigma_{i})=\frac{\exp(-1/2{\left(x-\mu_{i}\right)}^{T}\Sigma_{i}^{-1}(x-\mu_{i}))}{{\left(2\pi \right)}^{d/2}|\Sigma_{i}{|}^{1/2}}$$

$\theta ={\left\{\alpha_{k},\;\mu_{k},\;\Sigma_{k}\right\}}_{k=1}^{K}$ are iteratively updated based on Expectation-Maximization (EM) criterion to maximize the overall likelihood. GMM can provide the posterior probability of each symbol belonging to each cluster, which has advantages over other clustering methods, as it can serve as a measure of clustering confidence and provide valuable guidance for subsequent decision-making or training processes. Definition of posterior probability is shown as the following equation:

(19) $$\gamma_{ik}=\frac{\alpha_{k}N(x_{i}|\mu_{k},\Sigma_{k})}{\sum_{j=1}^{K}\alpha_{j}N(x_{i}|\mu_{j},\Sigma_{j})}$$

where $\gamma_{ik}$ is the posterior probability that sample $x_{i}$ belongs to the $k^{th}$ Gaussian component [48,49,50,51].

To ensure the reliability of the pseudo-labels, a confidence-based threshold is introduced. Only samples with sufficiently high posterior probability are retained during training updates, which helps suppress the influence of noise-induced ambiguous samples. This enables statistical alignment between the network output and the expected constellation geometry.

Compared with supervised DB-CNN, the proposed framework eliminates the dependency on ideal symbols, making it particularly suited to real-world communication scenarios where channel models are unknown or ground-truth is inaccessible. In environments affected by nonlinear distortion and hardware impairment, the proposed model optimizes itself through output-driven self-supervision, forming a closed-loop learning paradigm. This integration of statistical clustering and unsupervised trainable filtering establishes a viable and adaptable paradigm for building future neural-enhanced communication receivers.

## 3. Experimental Setup and Digital Signal Processing

### 3.1. Experimental Setup

**Figure 3:**
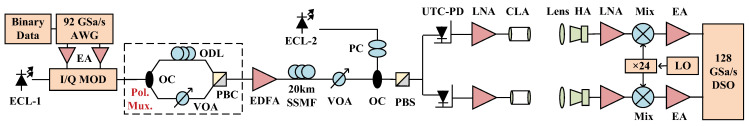
Experimental setup of 300-GHz PDM-MIMO THz system over the distance of 200 m. ECL: external cavity laser, AWG: arbitrary waveform generator, I/Q MOD: I/Q modulator, OC: optical coupler, ODL: optical delay line, VOA: variable optical attenuator, PBC: polarization beam combiner, EDFA: erbium-doped fiber amplifier, SSMF: standard single-mode fiber, PC: polarization controller, PBS: polarization beam splitter, UTC-PD: uni-traveling carrier photodiode, LNA: low noise amplifier, CLA: cylindrical lens antenna, HA: horn antenna, EA: electrical amplifier, LO: local oscillator, DSO: digital storage oscilloscope.

The experimental setup of the photonic-assisted THz PDM-MIMO system is shown in Figure 3. The transmitter includes an optical signal transmitter module and optical to radio frequency (RF) signal transformation module.

The optical signal transmitter module begins with a 1549.32-nm external cavity laser-1 (ECL-1) operating at 14 dBm as the optical carrier. The carrier is modulated with a baseband electrical QPSK signal generated by a 92-GSa/s arbitrary waveform generator (AWG) through an I/Q modulator. The modulated signal then passes through a polarization multiplexer. Inside the polarization multiplexer, the optical coupler (OC) first splits the light wave, and after data decorrelation operated by an optical delay line (ODL) and dual-arm power control via a variable optical attenuator (VOA), the polarization beam combiner (PBC) combines the signals with two orthogonal polarizations to generate a PDM-QPSK signal. This PDM-QPSK signal is then transmitted over a 20-km standard single-mode fiber (SSMF).

After compensating for link loss with an erbium-doped fiber amplifier (EDFA), the optical power is continuously adjusted via a VOA. The signal is then optically coupled with a local oscillator (LO) light emitted by the 1551.72-nm ECL-2, generating the carrier frequency of 300 GHz. Before coupling, a polarization controller (PC) is used to adjust the polarization of LO light, ensuring that it remains in a stable incoherent state relative to the signal. At the final stage, the pair of 300-GHz dual-polarized wireless signals are separated by a polarization beam splitter (PBS). Each signal is then detected by an UTC-PD, which symbolizes the optical-RF signal transformation session. Finally, dual-polarized signals are amplified by two 22-dB gain low noise amplifiers (LNAs), and transmitted via 48-dBi cylindrical lens antennas (CLAs).

**Figure 4:**
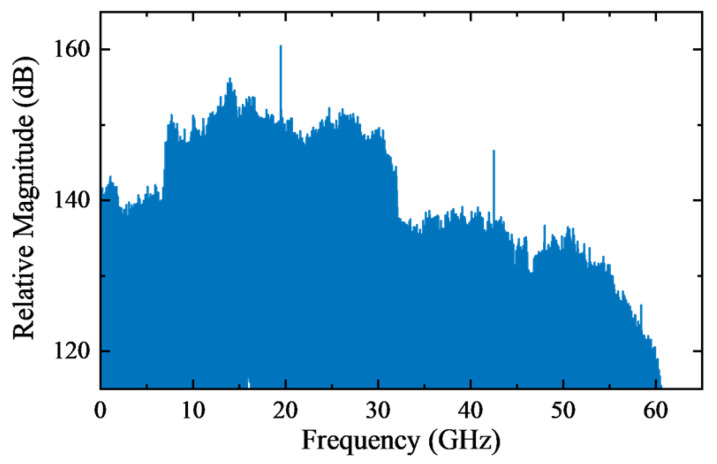
Spectrum of the received IF signal.

At the receiver, a pair of 30-cm diameter polytetrafluoroethylene (PTFE) lenses with a gain of 40 dBi, each equipped with a THz horn antenna (HA) with a 26-dBi gain, are used for reception. Subsequently, a pair of LNAs with a 22-dB gain are used to compensate for the power loss. The generated RF signals by an 11.6846-GHz RF source are fed into a ×24 multiplier, and then combine the received signals with two mixers, forming the down-conversion module. This operation generates an intermediate frequency (IF) signal at 19.57 GHz (300 − 11.6846 × 24 = 19.57). The IF spectrum is shown in Figure 4. Finally, the two IF signals are amplified by two 35-dB gain electrical amplifiers (EAs) and captured by a 128-GSa/s digital storage oscilloscope (DSO) with a 59-GHz bandwidth for offline DSP.

The whole terahertz wireless communication system was implemented through a line-of-sight (LOS) channel of 200 m. The experiment was carried out in sunny weather, with the temperature varying between 23 °C and 28 °C, and the relative humidity maintained at 40%. The free space path loss (FSPL) is about 128.1 dB and the gas attenuation is about 0.96 dB. Specifically, key parameters of the hardware setup are listed in Table 1.

### 3.2. Digital Signal Processing

**Figure 5:**
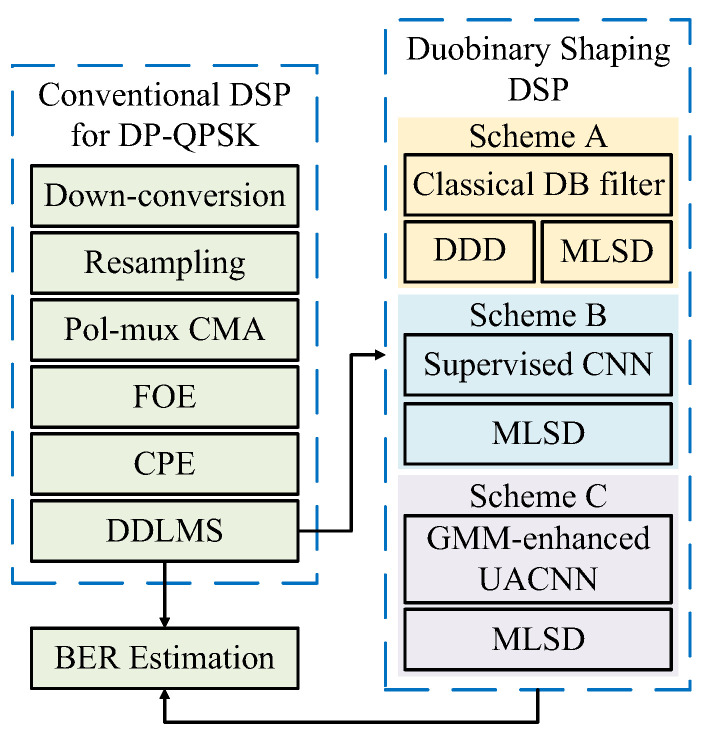
Offline DSP procedure at the receiver. Scheme A applies a classical DB filter followed by DDD and MLSD. Scheme B employs a supervised CNN equalizer before MLSD, and Scheme C adopts the proposed GMM-enhanced DB-UACNN equalizer followed by MLSD for final detection. Pol-mux CMA: polarization-multiplexed constant modulus algorithm, FOE: frequency offset estimation, CPE: carrier phase estimation, DDLMS: decision-directed least mean squares, DDD: direct differential decoder, MLSD: maximum likelihood sequence detection.

At the transmitter, a binary bit stream is first modulated into QPSK symbols. The modulated signal is then up-sampled with 2 samples per symbol (SPS) and shaped using a root raised cosine (RRC) filter with a roll-off factor of α=0.01. After wireless transmission over a distance of 200 m, PDM-QPSK signals undergo offline DSP as illustrated in Figure 5.

The received IF signals are down-converted to transform into baseband signals. Given the DSO sampling rate of 100 GSa/s, the baseband signals are resampled to 50 GSa/s, and fed into a T/2-spaced polarization-multiplexed constant modulus algorithm (Pol-mux CMA) as linear equalization. CMA helps mitigate inter-polarization crosstalk and channel noise, thus blindly recovering the two independent QPSK signals. After equalization, the dual-polarized signals pass through the FFT-based frequency offset compensation (FOE) and carrier phase compensation (CPE) using the blind phase search–maximum likelihood (BPS-ML) method. After carrier recovery, signals go through decision-directed least mean squares (DDLMS) algorithm in order to mitigate residual noise. Algorithms listed above are within conventional DSP for QPSK signals.

To address the limitation of high-frequency noise, three schemes are proposed and compared by each other. Scheme A uses a classical DB filter and 3-level duobinary signals are decoded by two methods, which are direct differential decoding (DDD) and MLSD. Scheme B replace the classical DB filter with a supervised DB-CNN. The output of CNN is decoded to QPSK signals through MLSD. It is noted that both the training and validation sets account for 50% of the data segment. Scheme C adopts GMM-enhanced DB-UACNN. The output of UACNN is also decoded by MLSD. By using grid search, parameters of GMM-enhanced DB-UACNN are listed in Table 2, as well as the process of the algorithms. The proposed network employs a shallow two-layer CNN with limited kernel sizes, resulting in low complexity comparable to FIR filtering. The training stage is conducted offline, and real-time operation only requires forward inference. From an operation-count perspective, the conventional DB filter represents the lowest-complexity baseline. For complex-valued signals, it requires only two real additions per output sample and no multiplications, resulting in linear complexity with an extremely small constant factor. The proposed DB-UACNN also exhibits linear time complexity of O(N), but with a larger yet fixed constant factor. During inference, the two-layer grouped convolution structure requires approximately two (taps1 + taps2) real multiplications per sample, together with a comparable number of real additions, while the nonlinear activation introduces an additional element-wise cost that also scales linearly with the signal length. With the parameters used in this work, this corresponds to roughly 448 real multiplications per sample, which remains moderate compared to high-order nonlinear equalizers. Also, the inference runtime was measured on a laptop equipped with an NVIDIA GeForce RTX 5070 GPU. After offline training, only forward inference was performed using fixed network parameters. Over 1000 repeated runs, the measured average inference time was 4.928 ms, with a variance of 0.480676 ms^2^, indicating stable and consistent runtime behavior. These results demonstrate the DB-UACNN achieves a favorable trade-off between complexity and performance. As a result, the proposed method can be readily integrated into existing receiver DSP chains, demonstrating strong potential for real-time implementation.

Finally, the performance is analyzed through BER and SNR. The recovered duobinary-shaped signal is compared with the conventional QPSK signal. The advantages of conventional DB filter and MLSD, as well as the performance comparison of the two proposed DB neural networks are illustrated in the next section.

## 4. Results and Discussion

### 4.1. Performance of Conventional DB Filter and MLSD

**Figure 6:**
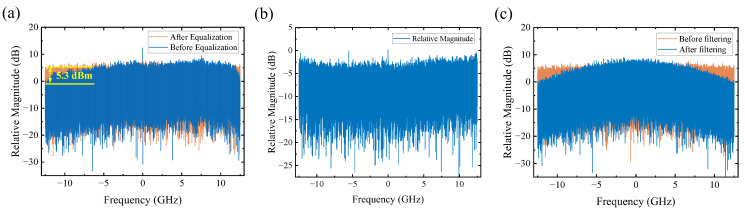
Spectrum of (**a**) signals before and after linear equalization, (**b**) noise of signals after linear equalization, and (**c**) signals before and after duobinary shaping.

As illustrated in Figure 6a, the received signal exhibits noticeable attenuation in the high-frequency components. After applying linear equalization using the PDM-CMA algorithm, the normalized power at high-frequency is compensated by approximately 5.3 dB. However, it amplifies the noise in the high-frequency region in Figure 6b. Figure 6c demonstrates the strong filtering capability of the classical DB filter defined by (1).

**Figure 7:**
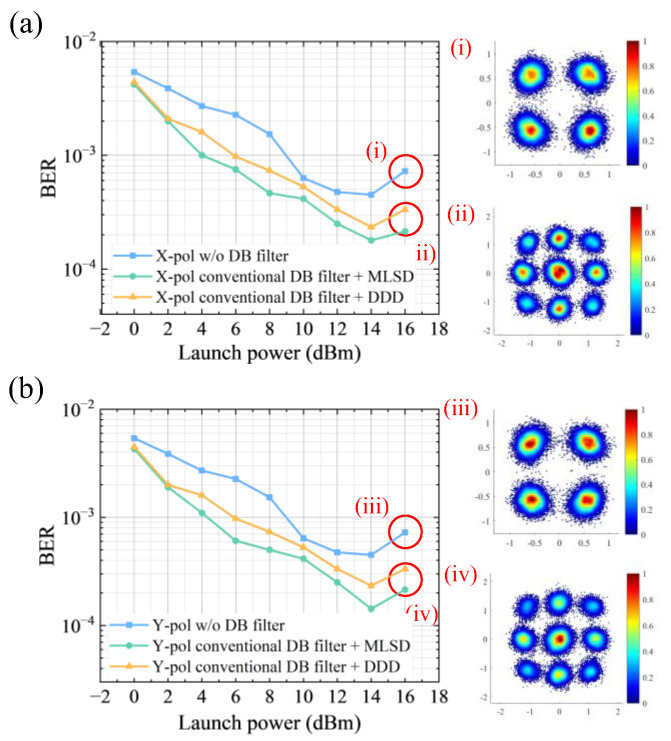
BER versus input optical power into UTC-PD for (**a**) X-polarized and (**b**) Y-polarized 25-GBaud QPSK signals in the 300-GHz terahertz wireless transmission system over 200 m, compared with conventional DB filter with MLSD and DDD, and a baseline without DB filtering. Subfigures (i) and (iii) donates the QPSK signals in both polarizations, while (ii) and (iv) donates the DB signals after the conventional DB filter.

From Figure 7, in both polarization channels, the system without classical DB filter (blue curve) consistently exhibits the worst BER throughout the entire optical input power into UPC-PD, indicating that the DB filters are the key components of improving the system performance. It is notable that the higher BER observed at 16 dBm compared to 14 dBm is because the system operated in the nonlinear regime of the optical devices. In contrast, the conventional DB filter (green and yellow curves) significantly improves performance. Figure 7i,iii shows constellations of QPSK signals. Duobinary signals generated by classical DB filters are shown in Figure 7ii,iv.

When comparing the detection strategies, the combination of the DB filter with MLSD (green curve) consistently outperforms DDD (orange curve), especially at launch powers relatively higher (4–16 dB). It can be explained as MLSD offers better noise suppression and ISI resilience. DDD, in contrast, relies solely on single-symbol decisions and is more susceptible to noise and distortion, indicating limited robustness.

However, as is elaborated before in (16), MLSD becomes more suboptimal under the system. As a result, the overall detection robustness is decreased, which is depicted in the BER curves as reduced smoothness in medium launch power and insufficiently obvious advantage over DDD in launch power of 0–2 dBm. As a result, more adaptive filtering strategies are required to improve resilience and generalization performance.

### 4.2. Comparison of Supervised DB-CNN and GMM-Enhanced DB-UACNN

**Figure 8:**
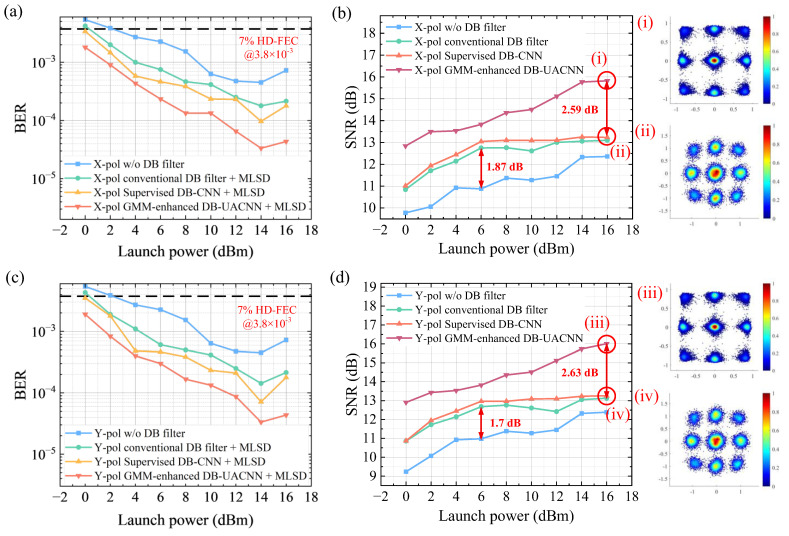
Comparison of BER and SNR performance under varying launch power. Specifically, BER versus launch power across different DB schemes are demonstrated in (**a**) X-polarization and (**c**) Y-polarization, SNR versus launch power across different DB schemes are demonstrated in (**b**) X-polarization and (**d**) Y-polarization. Subfigures (i) and (iii) donates output signals of GMM-enhanced DB-UACNN in both polarizations, while (ii) and (iv) donates signals after supervised DB-CNN.

Figure 8 systematically compares three DB filtering schemes in terms of communication performance in X- and Y-polarized channels, evaluating both BER and SNR performances. The three schemes include a conventional DB filter, a supervised DB-CNN-enhanced filter, and an unsupervised GMM-enhanced DB-UACNN filter. They are compared to each other and a baseline scheme without DB filtering. It is worth noting that the comparison among the baseline QPSK scheme, classical DB filtering, MLSD, supervised DB-CNN, and GMM-enhanced DB-UACNN constitutes an ablation analysis in Figure 7 and Figure 8. Specifically, the contribution of duobinary shaping is isolated by comparing the baseline QPSK scheme against conventional DB filtering + DDD scheme. The impact of MLSD detection is evaluated by comparing DB signals recovered using DDD and MLSD. The role of CNN-based enhancement is isolated by comparing the classical DB filter and the supervised DB-CNN, where both schemes employ MLSD to ensure fair detection. Finally, the effect of GMM-based unsupervised learning is quantified by comparing the supervised DB-CNN and the proposed DB-UACNN.

As shown in Figure 8a,c, although the conventional DB filter followed by MLSD scheme (green curve) offers improvement over the unfiltered QPSK baseline, it fails to adequately solve channel-induced nonlinearities. In both polarizations, the GMM-enhanced DB-UACNN+MLSD scheme (red curve) demonstrates a superior BER performance compared to all other DB filter schemes.

**Figure 9:**
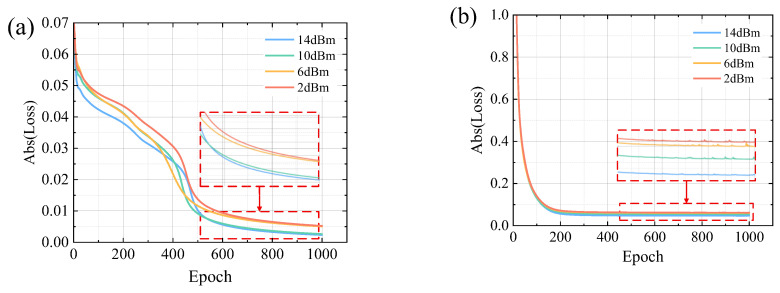
Loss Convergence through epochs of (**a**) GMM-enhanced DB-UACNN and (**b**) supervised DB-CNN model.

The supervised DB-CNN + MLSD scheme (yellow curve) also outperforms the conventional DB filter, but fails to have as well performance as the GMM-enhanced DB-UACNN. It is attributed to the fact that supervised learning expects the model to generalize well to unknown test data. However, its performance degrades with a mismatch between training and testing data. In contrast, unsupervised learning directly optimizes on the target input, achieving strong sample-specific adaptability. In particular, the proposed GMM-based unsupervised loss function leverages high-confidence clustering to generate pseudo-labels, enabling training to approach the effectiveness of supervised methods. As is shown in Figure 9, we compare loss function of the test data during the training. Figure 9a shows the training loss curves of the GMM-enhanced DB-UACNN under various launch powers, demonstrating stable convergence with consistent small decreases in loss at all power levels. In contrast, Figure 9b presents loss curves of the supervised DB-CNN, where the loss curves reach a plateau after early-stage convergence and shows fluctuation.

Figure 8b,d also present the SNR comparison for the four DSP schemes. Subfigures show the constellation of output of DB-UACNN in Figure 8i,iii and supervised DB-CNN in Figure 8ii,iv in both polarizations. The GMM-enhanced DB-UACNN consistently achieves the highest SNR gains, demonstrating its capability in effectively mitigating nonlinear noise and ISI. The supervised CNN performs second- best. In contrast, both the conventional DB and the unshaped schemes yield significantly lower SNR, especially under medium-to-high power conditions where improvements are limited.

Additionally, the SNR shows better improvement in higher launch power of UACNN. One reason is that it offers strong data adaptability by training directly on the received signal without requiring labels, enabling more effective compensation under severe nonlinear distortions at high launch powers. Additionally, clearer amplitude clustering at higher power improves the accuracy of GMM-based pseudo-labels, leading to more reliable loss convergence and enhanced robustness against distortion. It is worth noting that the proposed DB-UACNN exhibits low sensitivity to key hyperparameters, such as the GMM tolerance and the confidence threshold used for pseudo-label selection. In our experiments, varying these parameters within reasonable ranges resulted in negligible differences in BER performance. It suggests that the proposed framework does not rely on fine-tuned hyperparameter settings but instead demonstrates robust performance within a practical parameter range.

It is noted that both NN-enhanced DB filtering schemes consistently achieve BERs below the 7% HD-FEC threshold across all measured launch powers. While advanced channel coding schemes can further improve system-level performance [52], the pre-FEC BER metric adopted in this work provides a coding-independent evaluation of the proposed DSP and equalization framework. The duobinary shaping achieves up to a 1.87 dB and 1.70 dB SNR gain in two polarizations, respectively. The proposed GMM-enhanced DB-UACNN achieves an additional SNR gain of up to 2.59 dB and 2.63 dB over conventional DB filters, respectively.

Although differential decoding methods, such as DDD, are attractive due to their low implementation complexity, we find it hard to be adopted in the post-processing of neural network outputs. Instead, MLSD is often preferred. This preference arises for several key reasons: First, DDD relies heavily on the accuracy of the initial reference symbol and requires precise amplitude scaling. In neural network-based systems, the learned weights often introduce amplitude changes. As a result, inaccurate scaling between consecutive symbols can easily lead to catastrophic decoding failures. Secondly, error propagation is a significant concern. Even if the neural network successfully restores the signal to near-ideal constellation points, a single symbol decoding error can adversely affect the subsequent symbol. This “error chaining” effect reduces the robustness of differential decoding. Although MLSD entails higher complexity, it is generally a more reliable choice in NN-based systems where uncertainty and noise persist in the output.

### 4.3. Comparison of Neural and Model-Based Equalization Schemes

**Figure 10:**
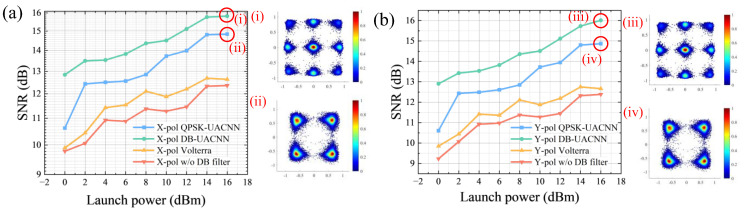
Comparison of SNR performance under varying launch power in (**a**) X- and (**b**) Y-polarization of DB-UACNN and QPSK-UACNN models. Subfigures (i) and (iii) donates signals of DB-UACNN in both polarizations, while (ii) and (iv) donates signals after QPSK-UACNN.

The SNR versus launch power curves of both polarizations is illustrated in Figure 10.

It demonstrates that the UACNN model whose target output is duobinary signals consistently outperforms its counterpart with a target of conventional QPSK signals. Figure 10i,iii is the DB-UACNN output of both polarizations, while Figure 10ii,iv is the output of the QPSK-UACNN. Specifically, a stable margin of approximately 1 dB across all power levels is achieved. It may because QPSK-DBCNN equalizers trained directly on QPSK symbols focus on symbol recovery, but this is not targeted to bandwidth-limited systems. This highlights the robustness of DB filters in unsupervised learning frameworks.

The proposed DB-UACNN is also compared with a conventional third-order Volterra nonlinear filter under different launch powers [53]. Note that the Volterra filter can compensate for third-order nonlinear noise, where 50% of the original data sequence is utilized for training. As shown in Figure 10, the DB-UACNN consistently achieves higher SNR than the Volterra-based equalizer across the entire launch power range. Conventional Volterra filters rely on predefined polynomial structures and fixed nonlinear orders, which limits their ability to adapt to bandwidth-limited and non-Gaussian channel conditions.

The experimental results demonstrate that the proposed method consistently achieves the best performance in both polarization channels across the full launch-power range. Its advantage becomes more evident at medium-to-high launch powers, indicating stronger robustness against practical nonlinear distortion and noise enhancement compared with conventional DB filtering and supervised DB-CNN. Although the proposed scheme introduces higher computational cost than the classical DB filter, the observed performance gains confirm that the additional complexity is justified and effective in improving receiver robustness.

## 5. Conclusions

In this work, we propose an offline DSP including duobinary shaping and optimized GMM-enhanced DB-UACNN, which is implemented on a 300 GHz 2 × 2 MIMO photonic-aided terahertz wireless system over 200 m. To address the challenges of limited bandwidth and severe high-frequency attenuation, we introduce a receiver-side duobinary shaping scheme combined with MLSD, which significantly enhances system robustness and signal integrity. The duobinary shaping consistently achieves better performance compared to conventional QPSK signals. To further mitigate nonlinear noise and improve adaptability under unknown channel conditions, we propose an unsupervised GMM-enhanced DB-UACNN model, which eliminates the dependency on labeled training data by leveraging soft clustering and pseudo-label generation. Experimental results show that this approach achieves an additional SNR gain over conventional DB filters, and consistently ensures BER performance below the 7% HD-FEC threshold at a 100 Gbit/s transmission rate.

While the proposed DB-UACNN demonstrates advantages above, its performance may degrade under extremely low-SNR scenarios caused by long transmission distances, atmospheric attenuation, or severe alignment errors, where GMM-based clustering becomes challenging. In addition, the proposed unsupervised adaptation assumes relatively stable channel statistics and known delay characteristics; therefore, rapidly time-varying channels may limit performance stability. These limitations define the boundaries of the proposed method.

Future work will primarily focus on real-time and hardware-oriented deployment of the proposed DB-UACNN. Owing to its shallow network structure and convolutional operations, the proposed method is well suited for hardware realization. Such studies will further assess the feasibility of integrating DB-UACNN into terahertz systems and bridging the gap between neural-enhanced DSP and deployable wireless systems.

## Figures and Tables

**Table 1:** Key parameters of components.

Device	Description	Model
AWG	Sampling rate: 92 GSa/s 3-dB bandwidth: 32 GHz	Keysight M8196
I/Q MOD	3-dB bandwidth: 25 GHz	FTM7961EX/301
UTC-PD	Operating band: 220–380 GHz DC responsivity: 0.22 A/W	NEL IOD-PMJ-13001
LNA	Operating band: 250–350 GHz typical gain: 22 dB noise figure: 12 dB P1dB: −5 dBm	RPG H-LNA 250–350
CLA	Operating band: 260–400 GHz directivity gain: 48.5 dBi 3-dB beamwidth (H/V pol.): 0.7°	Anteral LHA-HG-WR2.8
Lens	Operating band: 100–2000 GHz directivity gain: 40 dBi diameter: 30 cm focal length: 50 cm	PTFE-based
HA	Operating band: 260–400 GHz antenna gain: 26 dBi	Hanke RANT-260400-G26
Mix	Operating band: 260–400 GHz multiplication factor: ×24	OML C02.8DAS02
EA	Operating frequency: 0–50 GHz Gain: 35 dB noise figure: 4.5 dB	Fulai AT-LNA-0050-3505C
DSO	Sampling rate: 128 GSa/s 3-dB bandwidth: 59 GHz	Keysight UXR0594A

**Table 2:** Algorithmic summary of proposed DB-UACNN.

Stage	Process	
Initialization	Input data size	50,000
	Kernel 1 size	128
	Kernel 2 size	96
	Epochs	1000
	Learning rate	0.006
	Confidence threshold	0.99
	GMM initial means	$\left\{x+yj\;|\;x,y\in \{-1,0,1\}\right\}$
	Cluster numbers	9
	GMM tolerance	$10^{-6}$
Training Stage	$\begin{array}{l} {\left\{p_{i,k}\right\}}_{k=1}^{K},\mu_{1\times K}=\mathrm{GMM}\left(x_{i},K\right),\;i=1,\ldots ,N\\ for\;e=1,2,\ldots ,Epochs\;do\\ \;\;\;\;\;z^{\left(e\right)}=h_{\mathrm{CNN}}(\theta^{\left(e\right)};x)\\ \;\;\;\;\;p_{i,k}^{\left(e\right)}=\mathrm{Pr}\left(k|{\tilde{z}}_{i}^{\left(e\right)}\right)\\ \;\;\;\;\;S^{\left(e\right)}=\left\{i:\max_{k}p_{i,k}^{\left(e\right)}>\tau \right\}\\ \;\;\;\;\;L^{\left(e\right)}(\theta )=\sum_{i\in S^{\left(e\right)}}{\left\|{\tilde{z}}_{i}^{\left(e\right)}-\mu_{\arg\max_{k}p_{i,k}^{\left(e\right)}}\right\|}^{2}\\ \;\;\;\;\;\theta^{\left(e+1\right)}=\theta^{\left(e\right)}-\eta \nabla_{\theta }L^{\left(e\right)}(\theta )\\ end\;for \end{array}$	
Inference Stage	$z=h_{\mathrm{CNN}}(z;\theta^{\mathrm{Epochs}+1})$	

## Data Availability

The raw data supporting the conclusions of this article will be made available by the authors on request.
